# StearoylCoA Desaturase-5: A Novel Regulator of Neuronal Cell Proliferation and Differentiation

**DOI:** 10.1371/journal.pone.0039787

**Published:** 2012-06-22

**Authors:** Debora I. Sinner, Gretchun J. Kim, Gregory C. Henderson, R. Ariel Igal

**Affiliations:** 1 Section of Neonatology, Perinatal and Pulmonary Biology, Cincinnati Children's Medical Center, Cincinnati, Ohio, United States of America; 2 Department of Nutritional Sciences and Rutgers Center for Lipid Research, Rutgers, The State University of New Jersey, New Brunswick, New Jersey, United States of America; 3 Department of Exercise Science, and Rutgers Center for Lipid Research, Rutgers, The State University of New Jersey, New Brunswick, New Jersey, United States of America; National Cancer Center, Japan

## Abstract

Recent studies have demonstrated that human stearoylCoA desaturase-1 (SCD1), a Δ9-desaturase that converts saturated fatty acids (SFA) into monounsaturated fatty acids, controls the rate of lipogenesis, cell proliferation and tumorigenic capacity in cancer cells. However, the biological function of stearoylCoA desaturase-5 (SCD5), a second isoform of human SCD that is highly expressed in brain, as well as its potential role in human disease, remains unknown. In this study we report that the constitutive overexpression of human SCD5 in mouse Neuro2a cells, a widely used cell model of neuronal growth and differentiation, displayed a greater n-7 MUFA-to-SFA ratio in cell lipids compared to empty-vector transfected cells (controls). De novo synthesis of phosphatidylcholine and cholesterolesters was increased whereas phosphatidylethanolamine and triacylglycerol formation was reduced in SCD5-expressing cells with respect to their controls, suggesting a differential use of SCD5 products for lipogenic reactions. We also observed that SCD5 expression markedly accelerated the rate of cell proliferation and suppressed the induction of neurite outgrowth, a typical marker of neuronal differentiation, by retinoic acid indicating that the desaturase plays a key role in the mechanisms of cell division and differentiation. Critical signal transduction pathways that are known to modulate these processes, such epidermal growth factor receptor (EGFR)Akt/ERK and Wnt, were affected by SCD5 expression. Epidermal growth factor-induced phosphorylation of EGFR, Akt and ERK was markedly blunted in SCD5-expressing cells. Furthermore, the activity of canonical Wnt was reduced whereas the non-canonical Wnt was increased by the presence of SCD5 activity. Finally, SCD5 expression increased the secretion of recombinant Wnt5a, a non-canonical Wnt, whereas it reduced the cellular and secreted levels of canonical Wnt7b. Our data suggest that, by a coordinated modulation of key lipogenic pathways and transduction signaling cascades, SCD5 participates in the regulation of neuronal cell growth and differentiation.

## Introduction

As part of the development of the central nervous system, neuronal cells are required to coordinately expand their population and integrate a functional network by connecting through growing dendrites and axons, cell prolongations that are collectively denominated neurites. Neurite outgrowth is widely employed as a typical marker for assessing differentiation in cultured neuronal cells such as PC12 rat pheochromocitoma cells and Neuro-2a mouse neuroblastoma cells [1], [2]. Although the mechanisms by which neuronal cells control the timing of cell proliferation and differentiation are still poorly understood, animal and cell-based studies have shown that a number of extrinsic factors, including growth factors and cytokines, such as epidermal growth factor (EGF), platelet-derived growth factor, and brain-derived neurotrophic factor, have crucial influence on the functional fate of neuronal cells [2], [3]. The binding of these factors to plasma membrane receptors triggers the activation of central signal transduction cascades, including MAPK (ERK1/2), Akt and Src, which will initiate the transcriptional program needed for neuronal differentiation [3]–[5].

In addition to the aforementioned neurotrophic factors, Wnt proteins, a family of secreted proteins that modulates a myriad of cellular and organismal functions, including cellular proliferation, axis formation and organogenesis [6], [7], are key regulators of neuronal differentiation [8]. Binding of Wnt ligands to their receptor complex, consisting of members of Frizzled and low-density lipoprotein family LRP5 and LRP6, activates two main cascades of intracellular signals, the canonical β-catenin/TCF pathway, and the less understood non-canonical Wnt signaling which is independent of β-catenin. This signaling includes the planar cell polarity-convergent extension (PCP-CE) pathway, via Jun N-terminal kinase (jnk), Rho and Rac mediators [9], [10], and the Wnt/Calcium pathway which signals through Dvl to induce calcium influx and the activation of protein kinase C (PKC) and calcium/calmodulin-dependent protein kinase II (CaMKII) [11]. Wnt proteins have been shown to participate in the mechanisms of cell replication and differentiation in neurons, both in brain and in culture cells, by activating both signaling branches of Wnt pathways [8], but the mechanisms by which these signals regulate the timing of these processes is unclear.

The morphological changes that take place during the process of neuronal differentiation, such as neurite outgrowth, axon development and branching of neurite prolongations, also require a finely tuned regulation of lipid biosynthesis, especially the formation of new membrane phospholipids [12]. In mammalian cells, the biosynthesis of acyl-containing lipids employs saturated (SFA) and monounsaturated fatty acids (MUFA) as preferential substrates. The abundance of these fatty acids is determined, in great part, by the activity of StearoylCoA desaturases (SCD), key lipogenic enzymes that catalyze the conversion of SFA into MUFA. These fatty acid species, particularly MUFA, appear to be essential components for fetal brain development. Data from experiments performed in rats indicate that exogenous MUFA and SCD-derived MUFA are critical neurotrophic factors implicated in the modulation of axogenesis in brain [13], [14]. However, the potential implication of human SCDs in the mechanisms of neurogenesis and neuronal differentiation has remained understudied. Human tissues express two SCD variants, SCD1 and SCD5 [15]. Our lab and others have reported that SCD1, a Δ9-desaturase isoform present in most mammalian tissues, plays a key role in the regulation of lipogenesis, cell cycle and programmed cell death in human normal and cancer cells [16]–[20]. SCD5, a SCD isoform that was thought to be exclusive of primates but is also found in bovines, dogs and birds, is uniquely expressed in fetal brain, as well as in adult brain and pancreas [21]–[24], a distribution pattern that suggest that this enzyme may be implicated in critical neural functions. In this regard, it was recently reported that the expression of SCD5 mRNA was elevated in the brain of patients with Alzheimer's disease [25].

Unlike SCD1, the regulation and functional roles of SCD5 in human cells and tissues, especially in brain cell biology, have not been described to date. In the present study we show that constitutive expression of human SCD5 in mouse Neuro2a cells, a well-characterized cell model of neuronal differentiation [5], promotes a greater n-7MUFA-to-SFA ratio in total cell lipids and modifies the pattern of de novo synthesis of lipids. Concomitantly, SCD5 activity stimulated cell proliferation whereas it significantly suppressed the process of retinoic acid-induced differentiation of Neuro2a cells into mature neurons, suggesting a role for the desaturase in regulating the balance between cell expansion and differentiation of neuronal cells. We also provide evidence that signal transduction pathways that modulate these processes, such as epidermal growth factor receptor (EGFR)Akt/ERK and Wnt, are targeted for regulation by SCD5. Our data offer new insight into the role of SCD5 as a key factor in the coordinated regulation of lipogenic and signaling events that determine the biological fate of the neuronal cell.

## Materials and Methods

### Materials

Mouse neuroblastoma Neuro2a cells, WS-1 human normal fibroblasts, SH-SyS5 and human neuroblastoma cells were obtained from the American Type Culture Collection (Rockville, MD, USA). Dulbecco's modification of Eagle's medium (DMEM) with L-glutamine, MEM vitamin mixture and MEM nonessential amino acid solution were from Mediatech Cellgro (Manassas, VA, USA). Minimum Essential Medium (MEM) containing Earle's salts and L-glutamine, glucose free DMEM, phenol red free MEM, trypsin-EDTA solution and Lipofectamine™ 2000 transfection reagent were purchased from Invitrogen Corporation (Carlsbad, CA, USA). Heat-inactivated fetal bovine serum, crystal violet, protease and phosphatase inhibitor cocktail 2, fatty acid free bovine serum albumin, monoclonal anti β-actin antibody, and dimethyl sulphoxide (DMSO) were from Sigma-Aldrich (St. Louis, MO, USA). Nitrocellulose membrane, HPLC grade solvents, phosphate-buffered solution without calcium and magnesium and other cell culture supplies were obtained from Thermo Fisher Scientific (Pittsburgh, PA, USA). Pure fatty acid standards were purchased from Sigma-Aldrich or Nu-Check Prep (Elysian, MN, USA). Anti phospho-ERK1/2, antiphospho-EGFR (Tyr1068 and Tyr1086), antiphospho-Akt (S473) and total EGFR antibodies were from Cell Signaling Technology Inc (Danvers, MA, USA). GAPDH, Akt, ERK, HRP-conjugated anti mouse and anti rabbit IgG were from Santa Cruz Biotechnologies (Santa Cruz, CA, USA). Anti-V5 antibody was from Millipore (Billerica, MA). Luciferase and Renilla assay systems were obtained from Promega (Madison, WI). PathDetect c-Jun kinase reporter system was from Stratagene, Inc (Santa Clara, CA). D-[U-^ 14^C]glucose was purchased from American Radiolabeled Chemicals, Inc (St. Louis, MO, USA). Full-range rainbow molecular weight marker was from GE Healthcare Bio-Sciences Corp (Piscataway, NJ, USA). BCA Bradford protein assay kit and super signal West Pico chemiluminescent substrate were from Pierce (Rockford, IL, USA). Human SCD5 (∼37 kDa) was detected with a specific antibody kindly donated by Dr. Brent Rupnow, Bristol-Myers Squibb. This antibody was raised by immunizing rabbits with fusion proteins containing an SCD5-specific peptide sequence (MPGPATDAGKIPFCDAKEEIRAGLESSEGG) [20] and does not cross-react with the human SCD1 isoform (∼41 kDa) (Kim & Igal, unpublished observations).

### Cell culture

Unless otherwise stated, cells were cultured in DMEM supplemented with 10% FBS, penicillin (100 U/ml), streptomycin (10 µg/ml), 1% non essential amino acids and 1% MEM vitamin solution (growing medium). Cells were grown at 37°C, 5% CO_2_, and 100% humidity.

### Plasmids

The following constructs were previously used and described: pcDNA6 carrying the gene encoding human β-catenin with the S37A mutation [β-catenin (S37A)] [26], pcDNA6 V5 mSox4 pcDNA6 V5 mSox17, pcDNA6hTCF4, pcDNA V5 DEST hWnt7bV5 [27] was kindly provided by the Stem cell core CCHMC, pcDNA6 V5 mouse Wnt5a was provided by Dr Aaron Zorn, Cincinnati Children's Hospital Medical Center. TCF-luciferase reporter plasmids super8x TOP-flash and super8x FOP-flash [28] were generously provided by Randall Moon, University of Washington (Addgene plasmids 12456 and 12457). Human SCD5 cDNA cloned into a pcDNA™4/TO/myc-His expression vector was generously donated by Dr. Brent Rupnow, Bristol-Myers Squibb.

### Generation of human SCD5-expressing Neuro2a cells

Neuro2a cells, grown in 6-well plates up to 50% confluence, were washed twice with serum-free Opti-MEM and then transfected with 2.5 μg of either human SCD5 cDNA cloned in a pCDNA4/TO expression vector (Invitrogen), or empty plasmid (controls) plus 10 μl of LipofectAMINE 2000™ in 0.5 ml of Opti-MEM plus 20% FBS. After 5 h incubation, medium was replaced by 10%FBS MEM and transfected cells were grown for an additional 24 h. Then, positive SCD5-expressing cells (SCD5 cells) and empty vector-transfected cells (pCDNA4 cells) were selected by culturing the cells in 10% FBS, MEM containing zeocin (200 μg/ml) for 15 days. Presence of SCD5 protein was assessed by Western blot was described below.

### Cell lipid labeling, extraction and analysis

De novo synthesis of lipids in SCD5-expressing and control Neuro2a cells was assessed by the incorporation of [U-^14^C]glucose into cell lipids. Cells were incubated with 0.5 µCi/dish with [U-^14^C]glucose in 10%FBS DMEM for 16h. At the end of the labeling period, radioactive media was removed and cells were washed twice with 0.5% BSA in ice-cold PBS. Total cell lipids were then extracted following the procedure of Bligh & Dyer [29]. Total phospholipids and individual neutral lipids were separated by one-dimensional thin-layer chromatography (TLC) as described in Scaglia et al. [14]. Lipid spots on the TLC plate were stained with iodine vapors, and polar and neutral lipid fractions were scraped and their radioactivity counted in a liquid scintillation counter. The amount of [^14^C]tracer incorporated into lipids was normalized to cellular protein content of cells grown in parallel Petri dishes.

### Fatty acid analysis

Following organic extraction of total cell lipids and addition of 40 μg of heptadecanoic acid as internal standard, lipids were saponified to free fatty acids and then derivatized to their 2-nitrophenylhydrazide (2-NPH) derivatives as described in detail previously [30]. The derivatized compounds were then reconstituted with 150 μl methanol for analysis of fatty acids as their 2-NPH derivatives by high performance liquid chromatography (HPLC) using the method of Miwa et al. [31] as optimized by Henderson and Tuazon [30]. The relative abundance of 22 fatty acids in tissue lipid extracts were calculated as molar percentages based upon the following fatty acid external standards: Myristic acid (14∶0), Myristoleic acid (14:1n-5), Myristelaidic acid (*trans*-14:1n-5), Palmitic acid (16∶0), Palmitoleic acid (16:1n-7), Palmitelaidic acid (*trans*-16:1n-7), Stearic acid (18∶0), Oleic acid (18:1n-9), Elaidic acid (*trans*-18:1n-9), *cis*-Vaccenic acid (18:1n-7), *trans*-Vaccenic acid (*trans*-18:1n-7), Linoleic acid (18:2n-6), Linoelaidic acid (*trans, trans*-18:2n-6), α-Linolenic acid (18:3n-3), γ-Linolenic acid (18:3n-6), Arachidic acid (20∶0), Homo-γ-Linolenic acid (20:3n-6), Arachidonic acid (20:4n-6), Eicosapentaenoic acid (20:5n-3), Docosatetraenoic acid (22:4n-6), Docosapentaenoic acid (22:5n-3), Docosahexaenoic acid (22:6n-3).

### Induction of neuronal differentiation

Neuro2a cells, stable expressing SCD5 or empty expression plasmid, were plated at 5×10^4^ cells per 60-mm cell culture dish in 10%FBS DMEM and allowed to attach to the dish for 16 h. Media was then removed and cells were incubated with differentiation media (DMEM supplemented with 2%FBS and 20 µM *trans*-retinoic acid (RA)) for up to 96 h. A control group (undifferentiated cells) was maintained in 10%FBS DMEM. Cell bearing neurites equal to or longer than the cell diameter were considered to be differentiated cell. The experiment was performed in triplicate dishes and repeated three times with similar results. Data were expressed as percentage of total cells that were neurite-bearing.

### Determination of cell proliferation rate

Cells were seeded in 12 well plates (1.4×10^4^ cells per well). Twenty four hours later, the monolayers were rinsed with PBS and cells were grown in 10%FBS DMEM for up to 96 h. The media was changed every 48 h. Cellular proliferation was estimated by crystal violet staining following the procedure described by Menna et al. [32], with modifications. Briefly, cells were fixed with methanol, stained with 0.1% crystal violet in distilled water for 30 min and rinsed three times with water. The dye in the stained cells was solubilized in 10% methanol, 5% acetic acid solution and quantified by spectrophotometry at 580 nm. The optical density value of a blank well was subtracted in each case. The values at different time points were normalized to the data at 24 h after seeding to avoid differences due to disparity in cell adhesion efficiency or cell death.

### Determination of canonical and non-canonical Wnt activit

HEK 293T were plated into 24-well plates a day prior to transfection to yield density of 70% at transfection. Canonical Wnt signaling was monitored by TOP-flash assay. Cells were transfected with the following constructs: TOP-flash plasmid (50 ng), Fop flash plasmid (50 ng), which contains mutated binding sites for TCF and serves as a negative control of TOP-flash plasmid; renilla plasmid (50 ng) as a transfection control, the gene encoding a stabilized form of β-catenin (S37A) (100 ng), and the SCD5 construct (75 to 150 ng) by using Fugene 6 (Roche). The total amount of DNA was kept constant by adding empty-vector DNA. At 48 h post transfection cells were harvested and luciferase activity was measured as previously described [33]. Transfection efficiency was normalized using renilla luciferase activity from the control plasmid. Results are representative of three independent experiments. Non-canonical Wnt activity was measured by JNK activity using the PathDetect c-Jun kinase reporter system (Stratagene, Inc.). HEK 293T cells were transfected with the pFR-luc reporter plasmid (250 ng), the pGAL4-c-Jun-N-term vector (12.5 ng), and either pcDNA3/Wnt5a, (which serves as positive control by inducing JNK activity), pcDNA3/SCD5, or pcDNA3. The pGAL4DBD was used as a negative control of pGAL4-c-Jun- N-term vector since it does not contain activation domain. The pFC MEKK (12.5 ng) was used as positive control for the assay since it phosphorylates the transreporter thus inducing luciferase synthesis. Luciferase activity was measured as described before.

### Analysis of synthesis and secretion of Wnt proteins

In order to analyze the intracellular and secreted levels of Wnt5a (∼42 kDa) and Wnt7 b (∼39 kDa), Neuro2a cells overexpressing SCD5 (SCD5) and control Neu2a were plated into 24-well plates 24 hs prior to transfection and then were transiently transfected with Wnt tagged constructs (mWnt5aV5 or hWnt7myc). Groups of cells were transfected with either Sox17 or TCF cDNA and employed as controls of protein synthesis. Twelve hours post-transfection culture media was replaced with 150 µl serum free media. After 24 h incubation, cell media and cells were collected and processed for SDS-PAGE. Western blots were performed using anti V5, anti myc and anti GAPDH antibodies. Experiments were performed using three to four replicates and repeated at least twice.

### Immunoblotting

After corresponding treatments, cells were rinsed with ice cold PBS, scraped in cold hypotonic lysis buffer (20 mM Tris-HCl pH 7.5, 10 mM NaF, 1mM EDTA, plus protease and phosphatase inhibitor cocktails) and homogenized by sonication in ice-water bath. Fifty µg of total cellular proteins were resolved by SDS-PAGE and transferred onto a nitrocellulose membrane. After blocking, the membranes were incubated overnight at 4°C or for 2 h at room temperature with primary antibody. Horseradish peroxidase-conjugated secondary antibodies were used in 1∶10,000 dilutions. Proteins on the membrane were detected using a West Pico chemiluminescence detection kit and photographed with a ChemiDoc (BioRad) digital image system. Immunoblots were repeated at least three times with essentially the same results.

### Determination of cellular protein

Total cellular protein content was measured by Bradford method, using BSA as a standard.

### Statistical analysis

Results from a representative experiment with at least 3 samples per experimental group are presented as means ± S. D. Statistical significance of the data was determined by Student's t-test.

## Results

### Expression of human SCD5 gene in Neuro2a cells increases the n-7 MUFA-to-SFA ratio

**Figure 1:**
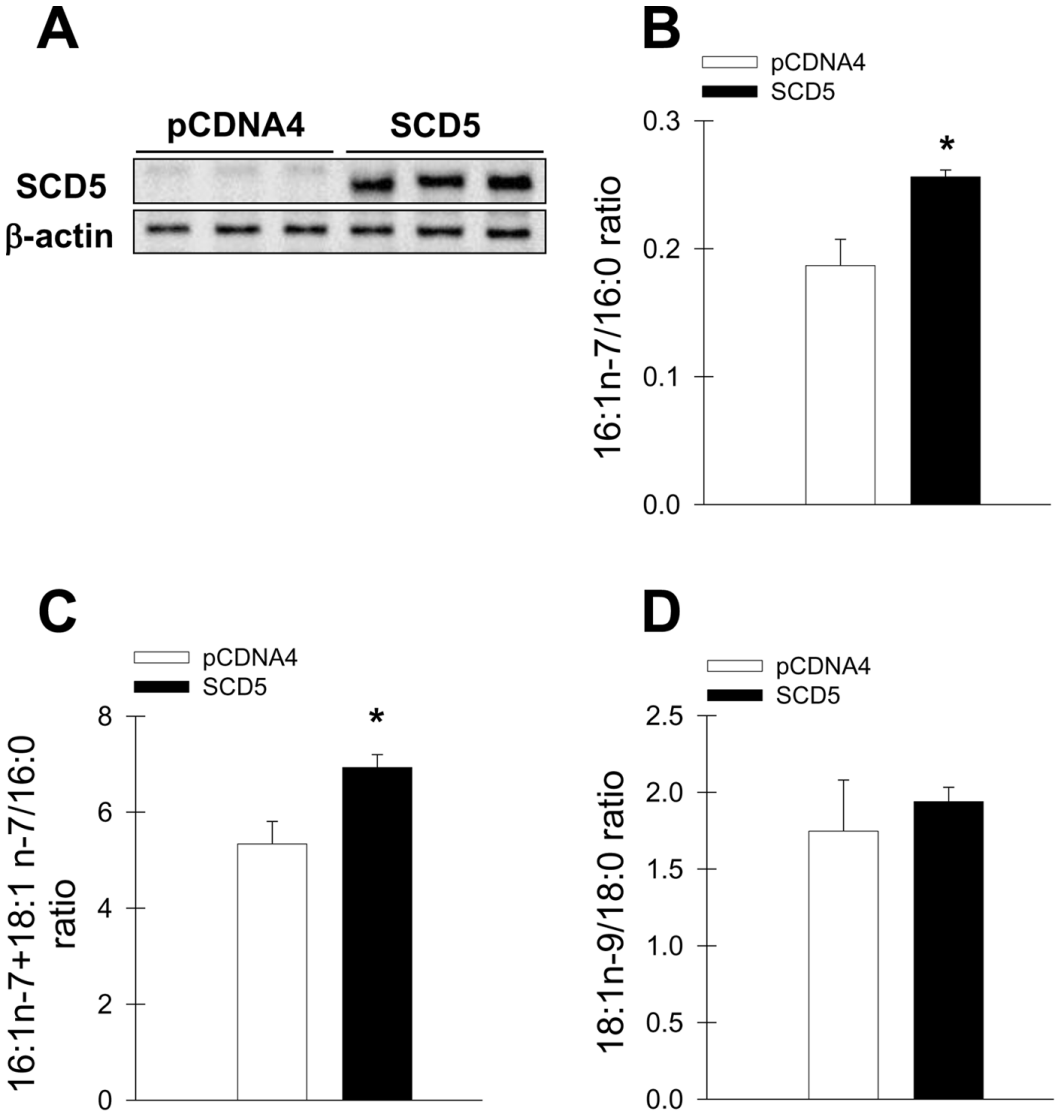
Overexpression of human SCD5 increases the ratio n-7 MUFA to SFA. Neuro2a cells were stably transfected with human SCD5 cDNA in a pCDNA4 expression plasmid (SCD5 cells) or with empty plasmid alone (pCDNA4 cells). Expression of SCD5 was determined by immunoblot analysis of cell homogenates (*A*). Fatty acids from total cell lipids were assessed by HPLC and ratios MUFA to SFA were calculated. *B*, palmitoleic acid (16:1n-7) to palmitic acid (16∶0); *C*, palmitoleic acid plus cis-vaccenic(18:1n-7) to palmitic acid (16∶0); D, oleic acid (18:1n-9) to stearic acid (18∶0). *, p<0.05 or less.

To begin assessing the metabolic and biological implications of SCD5 expression, we established a cell model of SCD5 expression in mouse neuroblastoma Neuro2a cells, which do not naturally express SCD5. This cell model serves a two-fold purpose: it allows us to investigate the physiological and metabolic roles of human SCD5, a SCD isoform that has remained unexplored; and also to determine a potential role of this desaturase in the processes of neuronal cell growth and differentiation. Thus, cells were stably transfected with SCD5 cDNA (SCD5 cells) or with empty pCDNA4 expression vector (pCDNA4 cells) and subjected to positive selection with puromycin for two weeks. As we expected, ectopic expression of SCD5 protein in transfected cells with the SCD5 construct (Figure 1A) was verified in cell homogenates by Western Blot analysis. The fatty acid analysis by HPLC suggests that the ectopic SCD5 was functionally active in the neuronal cells since expression of SCD5 markedly altered the balance MUFA-to-SFA, increasing the ratios n-7 palmitoleic acid (16∶1)/palmitic acid (16∶0) (Figure 1B) and cis-vaccenic acid (18∶1) + palmitoleic acid/palmitic acid by ∼20–25% (Figure 1C). The altered fatty acid ratios were the result of a significant increase in palmitoleic acid (mol% = 5.40±0.58, controls; 7.05±0.18, SCD5 cells, p<0.01 by Student's t test), and a parallel reduction, albeit slight, in palmitic acid (mol% = 28.69±0.25, controls; 27.31±0.25, SCD5 cells, p<0.03 by Student's t test). Levels of oleic acid (18∶0) and stearic acid were not significantly changed by SCD5 overexpression (mol% oleic acid = 27.22±3.28, controls; 28.47±0.22, SCD5; mol% stearic acid = 15.63±1.28, controls; 14.61±0.28, SCD5), which caused the balance between the n-9 MUFA/SFA to remain essentially unchanged (Figure 1D), suggesting that SCD5 expression in Neuro2a cells specifically induces the production of n-7 MUFA.

#### Alteration of glucose-mediated lipid synthesis by SCD5 expression

**Figure 2:**
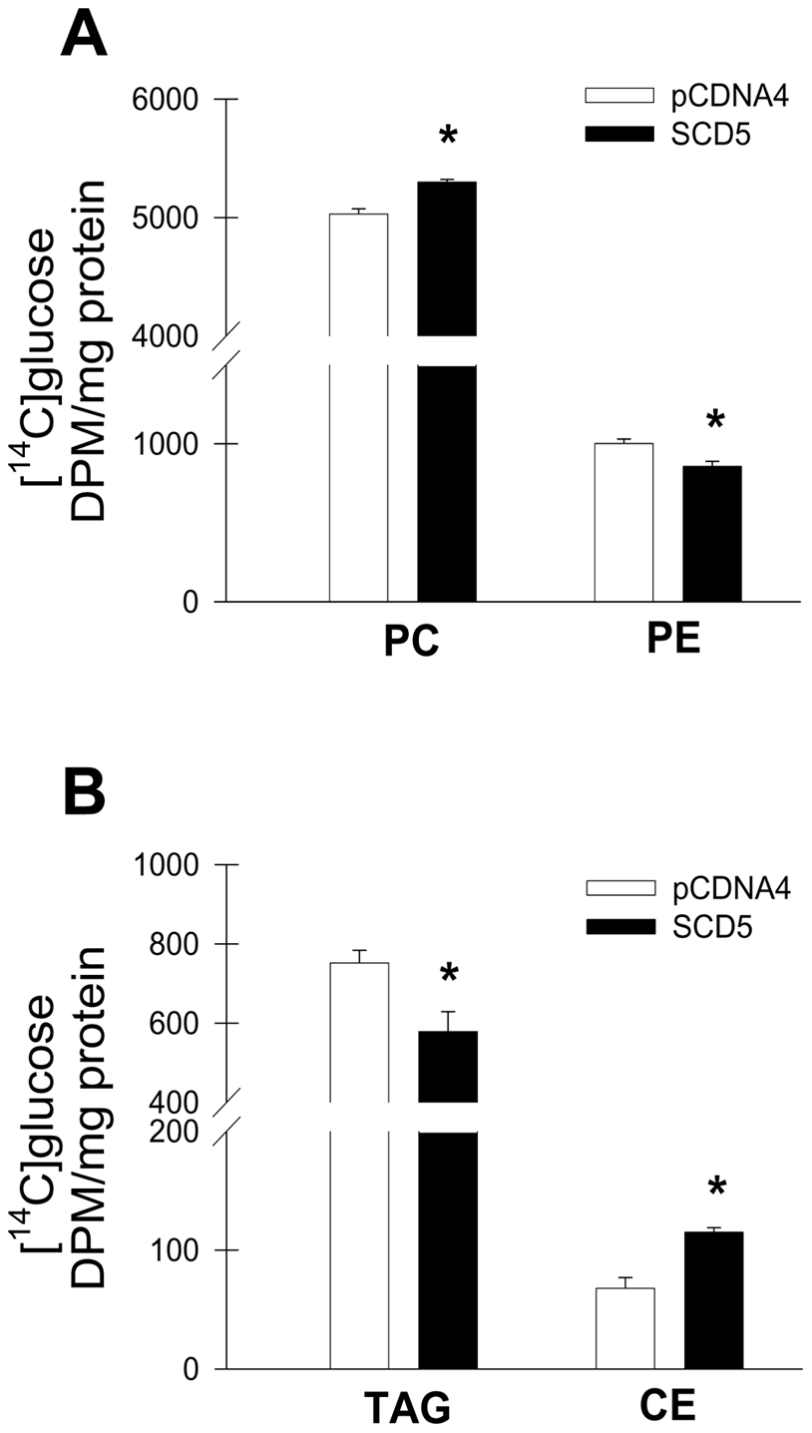
Expression of human SCD5 in Neuro2a cells modifies glucose-mediated synthesis of lipids. Neuro2a cells stably transfected with human SCD5 cDNA (SCD5 cells) or with empty plasmid (pCDNA4 cells) were incubated with [U-^14^C]glucose in 10%FBS DMEM for 16 h. Total lipids were then extracted and main polar and neutral [^14^C]labeled-lipids were separated by one-dimensional thin-layer chromatography, quantified by radiometric densitometry and normalized to protein concentration. *A*, phosphatidylcholine (PC) and phosphatidylethanolamine (PE); *B*, triacylglycerol (TAG) and cholesterolesters (CE). Values represent the mean ± SD of quadruplicate samples. *, p<0.05 or less vs control, by Student's t test.

Normal proliferating cells and cancer cells activates lipogenesis, particularly phospholipid formation, in order to cope with the metabolic requirements of cell proliferation [15], [33], [34]. In these cells, the degree of activation of SCD1 is directly linked to the rate of lipid synthesis [16], [17]. The participation of human SCD5 in the modulation of lipogenesis has not been addressed to date. In order to investigate the mechanisms of metabolic regulation by SCD5 in mammalian cells, we studied the effect of constitutive expression of SCD5 on glucose-mediated lipid biosynthesis in proliferating Neuro2a cells. Cells were incubated with [U-^14^C]glucose for 16 h and the levels of [^14^C]labeled lipids were determined. The incorporation of carbons from the [^14^C]glucose into total lipids was found unmodified by the expression of ectopic SCD5 (data not shown). [^14^C]label was mostly incorporated into phospholipids, particularly phosphatidylcholine (PC) and phosphatidylethanolamine (PE) (Figure 2A ), as well as in lower amounts in phosphatidylinositol and phosphatidylserine (not shown). Biosynthesis of PC was significantly, albeit slightly, elevated whereas PE levels were decreased in SCD5-cells compared to their controls. Neutral lipids triacylglycerol (TAG) and cholesterolesters (CE) represented a quantitatively minor fraction of [^14^C]labeled-lipids (Figure 2B). The incorporation of radiolabeled glucose into TAG was significantly reduced (∼20%), whereas the presence of [^14^C]tracer in CE was increased by ∼85% in SCD5-expressing cells with respect to controls. Taken together, these findings suggest that SCD5 activity participates in the control of de novo lipid synthesis in neuronal cells. Also, the observation that SCD5 expression induces a differential partition of [^14^C]glucose label into lipid species indicates that it affects the functional segregation of fatty acids into different lipogenic routes.

#### Expression of human SCD5 in Neuro2a cells reduces neuronal differentiation and increases cell proliferation

**Figure 3:**
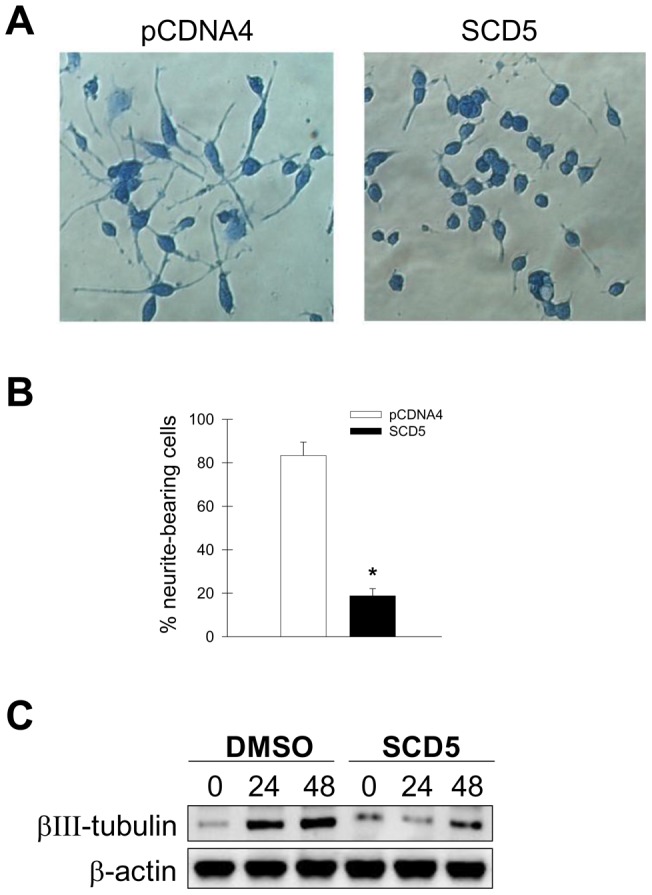
SCD5 activity blocks retinoic acid-induced differentiation of Neuro2a cells. SCD5-expressing Neuro2a cells and empty plasmid-carrying controls (pCDNA4) were seeded in 60-mm dishes and incubated in 10%FBS DMEM. Twenty-four hours later, media was removed and cells were incubated with differentiation media (DMEM supplemented with 2%FBS and10 µM retinoic acid) for 48 h. Cells were fixed, stained with coomassie brilliant blue proliferation and photographed in a phase-contrast microscope (*A*). *B*, percentage (%) of cells bearing one or more neurites equal to or longer than cell body diameter.

Regulation of lipid metabolism is critical for the balance between cell replication and differentiation that determine the fate of neuronal cells [5]. Given the fact that stably expressed SCD5 markedly alters fatty acid composition and lipid biosynthesis, we determined whether neuronal cell growth and differentiation were affected by these modifications in lipid metabolism. Neuro2a cells, expressing SCD5 and controls, were incubated in 2%FBS DMEM plus 10 µM RA for 96 h to promote neuronal maturation. A group of cells was placed in 10%FBS DMEM and they were considered undifferentiated controls. It was observed that induction of differentiation resulted in a significant development of neurites in pCDNA4 control cells (Figure 3A, left panel), with over 80% of cells displaying neurite outgrowth after a 48 h incubation (Figure 3B). Remarkably, in cells expressing human SCD5 neuritogenesis was notably suppressed (Figure 3A, right panel), with only ∼20% of cells showing neurite prolongations (Figure 3B). After 96h of differentiation, all plasmid-transfected control cells exhibited long branching neurite prolongations, whereas a majority of SCD5-cells showed fewer and shorter neurite outgrowths (data not shown). We also determined the presence of βIII-tubulin, a typical early marker of neuronal differentiation [35], upon incubation with 10 µM RA for 24 and 48 h. In absence of RA, SCD5 and control cells showed low levels of βIII-tubulin (Figure 3C). Twenty four hours after induction of differentiation, βIII-tubulin markedly increased in control cells, remaining elevated after 48 h of RA treatment. Although βIII-tubulin slightly increased in SCD5 cells at 24 and 48 h of differentiation, the levels of this protein were significantly more reduced than in control cells. Altogether, these results indicate the significant impact of SCD5 activity on the program of neuronal differentiation.

**Figure 4:**
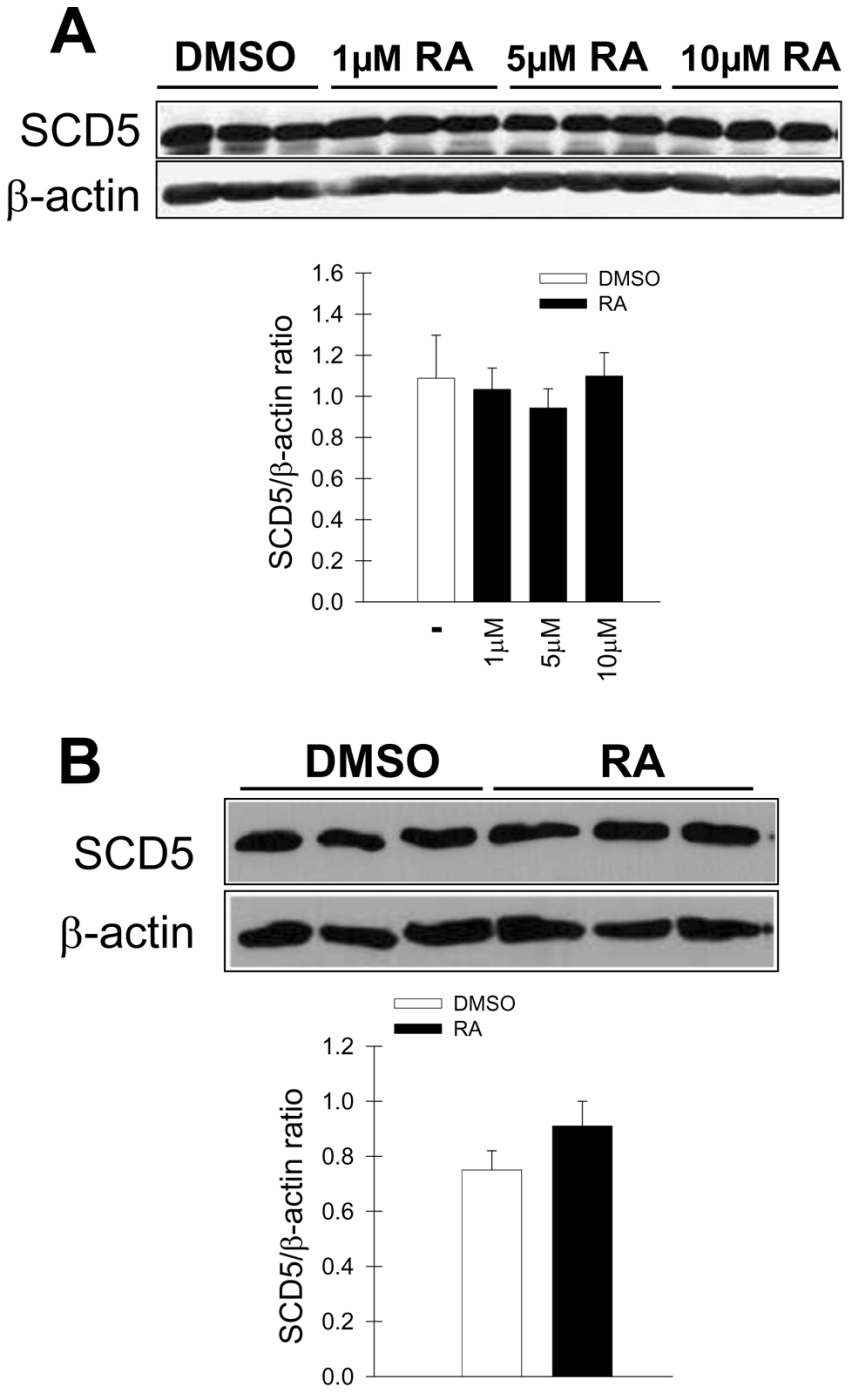
Effect of retinoic acid on the expression of SCD5 in human cells. WS-1 human normal skin fibroblasts were treated with increasing concentration of retinoic acid (RA) up to 10 µM or vehicle DMSO (0.1% by vol) for 24 h. SCD5 and β-actin in cell homogenates were detected by immunoblot analysis and were quantified by densitometry (*A*). SH-SY5Y human neuroblastoma cells were incubated with 20 µM RA or DMSO for 4 days and content of SCD5 protein was assessed by Western blot (*B*). Levels of SCD5 were normalized using β-actin values. Data represent the mean ± SD of triplicate samples. *, p<0.05 or less vs control, by Student's t test.

To further delineate the potential functional relationship between SCD5 and the program of neuronal differentiation, we examined whether retinoic acid would affect the levels of SCD5 in human cells. We observed that treatment with concentrations of retinoic acid for up to 10 µM did not modify the content of SCD5 enzyme in WS-1 normal human skin fibroblasts cells (Figure 4A). Incubation of SH-SY5Y human neuroblastoma cells with 10 µM RA for 96 h, a treatment that leads to induction of neuronal differentiation [36], also failed to change the levels of SCD5 protein in these cells (Figure 4B). Together with data from the differentiation experiments, these results suggest that although SCD5 expression may not be targeted by the differentiation program, the activity of the desaturase may be crucial for its execution.

**Figure 5:**
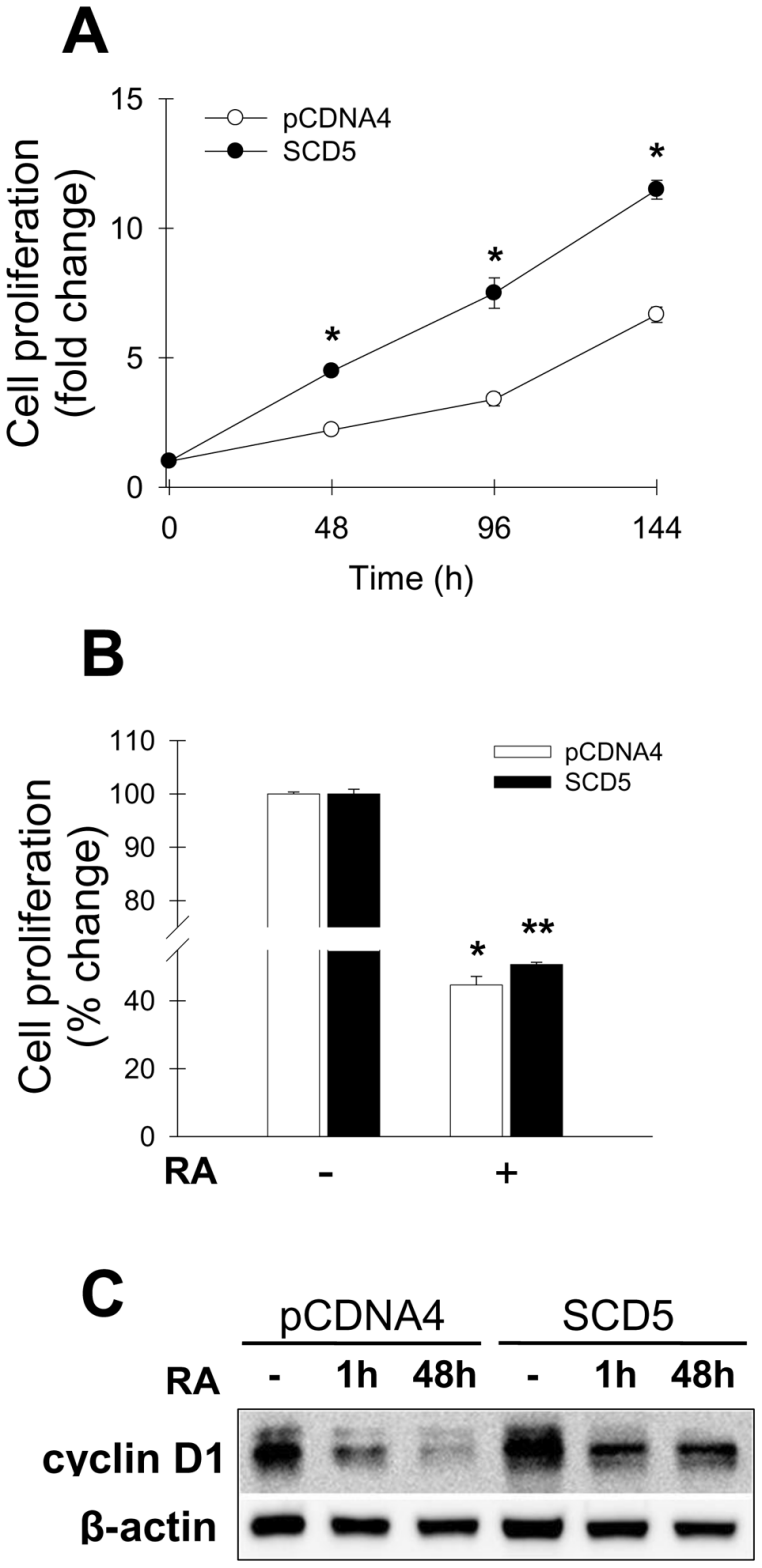
Expression of human SCD5 in Neuro2a cells increases cell proliferation. Effect of retinoic acid. Neuro2a cells stably transfected with human SCD5 cDNA (SCD5 cells) or with empty plasmid (pCDNA4 cells) were seeded in 12-well plates and grown for different time points up to 120 h (*A*). At each time point, cell proliferation was determined by Crystal violet assay as described in Materials and Methods. *B*, SCD5-expressing Neuro2a cells and their controls were incubated with 10 µM retinoic acid or DMSO vehicle for 48 h. Cell growth was estimated by Crystal violet staining method. Values represent the mean ± S.D. of triplicate determinations. *, p<0.05 or less vs control, by Student's t test. *C*, Western blot determination of cyclin D1 and β-actin levels in control (pCDNA4) and SCD5-expressing Neuro-2a cells incubated in serum-free DMEM with 0.1% BSA, in presence or absence of 10µM retinoic acid (RA), for 1 h or 48 h.

To further analyze whether the decreased differentiation rate in SCD5-overexpressing Neuro2a cells was accompanied by other modifications in the biological phenotype of these cells, empty plasmid-transfected control and SCD5 cells were tested for cell proliferation. The presence of SCD5 activity in Neuro2a cells resulted in a marked increase (80–100%) in the cellular proliferation rate (Figure 5A), strongly suggesting that the higher MUFA-to-SFA ratio in these cells propelled the mitogenic process. As expected, incubation of SCD5 expressors and their controls with 10 µM retinoic acid for 24 h induced a profound reduction in cell proliferation (Figure 5B), although this decreasing effect was attenuated, albeit slightly, in the SCD5-expressing cells as compared with controls. Interestingly, incubations with 10 µM RA for 1h and 48 h greatly reduced the levels of cyclin D1, a key protein that propels cell cycle [37], in control Neuro2a cells, but this decreasing effect was much more attenuated in SCD5-expressing cells (Figure 5C). Altogether, the results of these experiments indicate that SCD5 activity controls neuronal fate by suppressing neuronal differentiation while concomitantly activating cell proliferation, likely by accelerating the progression of cell cycle.

#### Expression of SCD5 alters the EGF-dependent signaling mechanism

**Figure 6:**
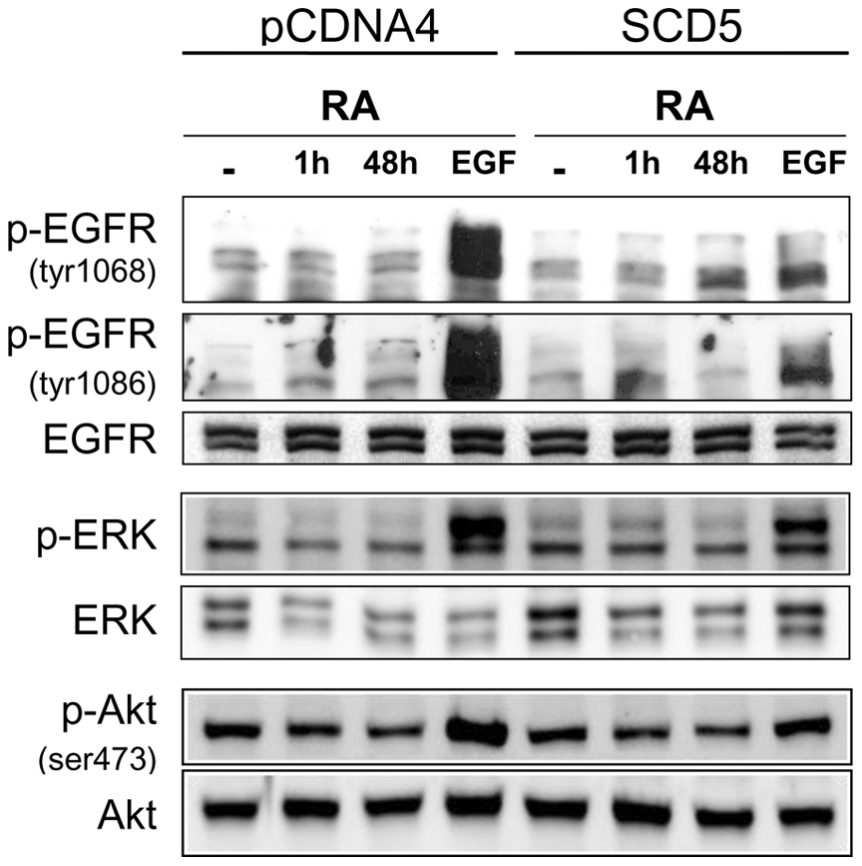
SCD5 expression impairs the activation of EGFR signaling cascade. Neuro2a cells, stably transfected with SCD5 cDNA (SCD5) or empty plasmid, were incubated in serum-free DMEM with 0.1BSA, in presence or absence of 10 µM retinoic acid (RA), for 1 h or 48 h. A group of cells incubated with RA for 48 h was stimulated with 100 ng/mL EGF for 5 minutes. Cells were harvested and homogenized and phosphorylation of EGFR, ERK, and Akt was determined by Western blot.

A key aspect of our hypothesis is that SCD5 activity, by producing specific changes in lipid molecular species, may modulate plasma membrane-resident signaling platforms that control cell proliferation and differentiation. As mentioned above, EGFRAkt/ERK signaling cascade is one of the most ubiquitous players in the mechanisms of neuronal growth and maturation [6]; thereby we analyzed the activation of this receptor in neuronal cells by assessing its phosphorylation upon binding of EGF. Induction of differentiation with retinoic acid has been shown to induce autophosphorylation of EGFR in Neuro-2a cells [12]. We incubated the cells, both expressing SCD5 or empty expression plasmid, with retinoic acid for 1 h and 48 h in serum-free DMEM and determined the phosphorylation of Tyr1068 and Tyr1086, two tyrosine residues that are critical for the functional activation of EGFR. As shown in Figure 6, retinoic acid did not affect the phosphorylation levels of Tyr residues at any incubation times in either cell group. However, when cells were stimulated with EGF for 5 minutes, a robust phosphorylation of Tyr1068 and Tyr1086 residues was observed in controls cells whereas SCD5-cells displayed lower levels of EGFR phosphorylation (Figure 6 ), suggesting that MUFA products of SCD5 activity downregulate the ligand-mediated activation of EGFR in neuronal cells.

Since phosphorylation of Tyr1068 and Tyr1086 are functionally linked to the activation of Akt and ERK signaling pathways, next we examined whether the blunted EGF-induced activation of EGFR seen in SCD5 expressors would be translated to its downstream effectors. Again, treatment with retinoic acid alone did not evoke changes in the phosphorylation status of either Akt or ERK proteins. Incubation with EGF, in turn, induced strong phosphorylation of both signaling proteins in pCDNA4-carrying control cells, but this effect was noticeably attenuated in SCD5-expressing cells (Figure 6). Altogether, these observations indicate that fatty acids produced by SCD5 activity, or lipids acylated by these fatty acids, negatively modulate the signaling platform EGFRAkt/ERK upon stimulation and that this regulatory effect may be implicated in the alterations in cell growth and differentiation observed in cells expressing SCD5.

#### Modulation of canonical and non-canonical Wnt activity by SCD5

**Figure 7:**
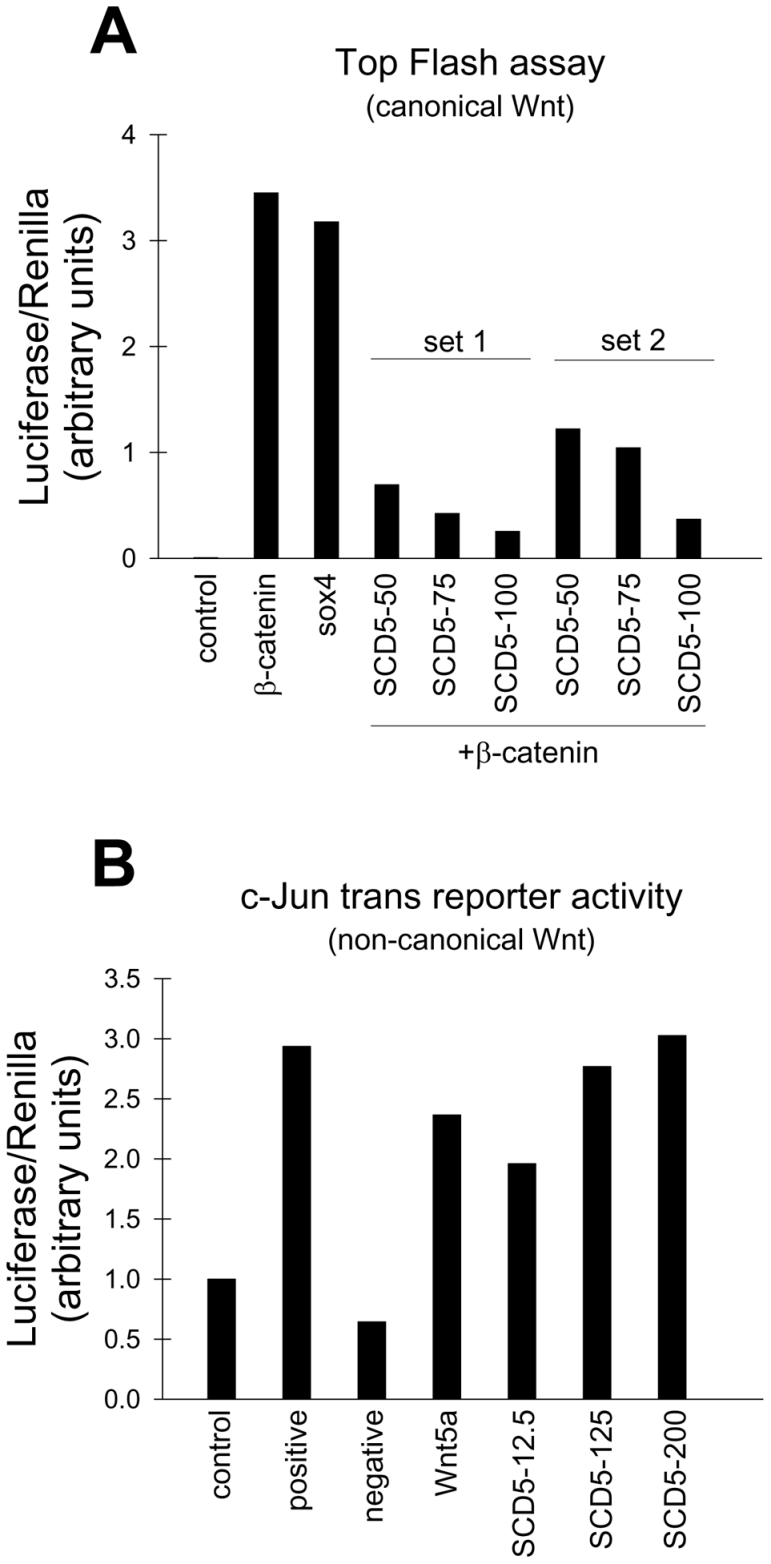
SCD5 modulates the activity of both canonical and non-canonical branches of Wnt signaling pathways. Canonical (*A*) and non-canonical (*B*) Wnt activity was assessed by TOP-Flash and c-jun trans-reporter assays, respectively. For these determinations, HEK 293T cells were transfected with indicated constructs. In the TOP-Flash assay, sox4 was used as a positive control. For the c-jun trans-reporter determination, in addition to the c-jun activator Wnt5a, pGAL4DBD, which does not contain the activation domain, and pFC MEKK, which activates the reporter through phosphorylation, were used as negative and positive controls, respectively. Forty eight hours post transfection cells were harvested and processed for luciferase assay. Values of c-Jun trans reporter assay are the average of duplicate samples. Variability of duplicates was less than 5%. All data are representative of three independent experiments.

Wnt proteins are also critical extrinsic factors that modulate neuronal cell expansion, as well as the entry in the differentiation program [38], [39]. Wnt proteins require posttranslational acylation with palmitic acid and palmitoleic acid in conserved cysteine and serine residues, respectively for optimal secretion and activity [40], [41]. Since we observed that SCD5 expression alters the balance between SFA and MUFA, it is conceivable that Wnt activity may be targeted for modulation by SCD5. To examine this hypothesis, wild-type HEK293 cells were co-transfected with cDNA encoding SCD5 or empty plasmid, plus the luciferase reporter constructs pTOP-flash or c-Jun trans reporter that monitor the activity of the canonical Wnt and non-canonical Wnt pathways, respectively. For the determination of TOP-Flash activity, Sox4 was employed as positive control for the assay system. As expected, expression of β-catenin stimulated canonical Wnt activity (Figure 7A). Expression of increasing amounts of SCD5 construct in cells promoted a progressive decrease in β-catenin-induced TOP-Flash activity (Figure 7A), indicating that the desaturase activity is able to modify canonical Wnt activation. Furthermore, increasing levels of transfected SCD5 induced a parallel increase in non-canonical Wnt reporter activity (Figure 7B) when compared to empty pCDNA plasmid-transfected cells. We take these results to suggest that SCD5 activity modulates both Wnt pathways in opposite directions, an effect that could be responsible for the alterations in cell proliferation, differentiation, or both, seen in Neuro-2a cells.

#### SCD5 activity controls the synthesis and secretion of Wnt7b and Wnt5a proteins

**Figure 8:**
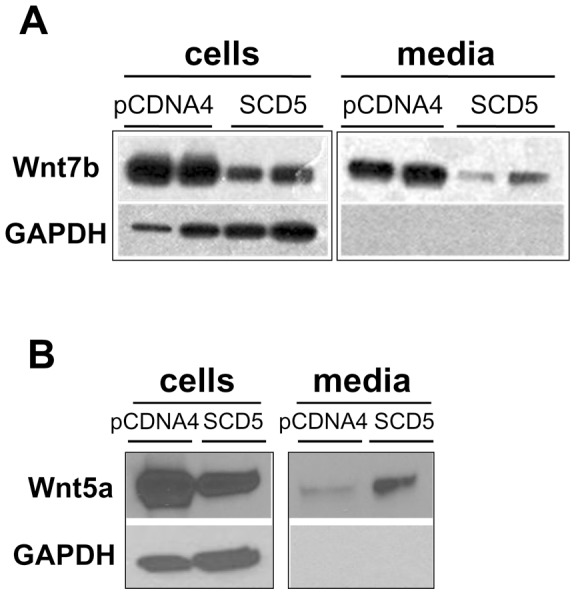
SCD5 reduces the secretion of canonical Wnt7b whereas it increases the secretion of non-canonical Wnt5a. To determine the synthesis and secretion of Wnt7b and Wnt5a, Neuro2a cells, both control (pcDNA4) and SCD5-overexpressing (SCD5), were transiently transfected with Wnt7b- and Wnt5a- tagged constructs. Twelve hours post-transfection, the culture media was replaced with serum-free media. After 24 h, cell media and cells were collected and processed for SDS-PAGE. Western blots were performed using anti V5 and anti GAPDH antibodies. This experiment is representative of three independent assays.

As mentioned above, fatty acid binding to Wnt residues is a critical feature for the secretion of these proteins. Recently, it was reported that a specific acylation conserved serine residue of Wnt3a with palmitoleic acid (16:1n-7) is a prerequisite step for the secretion of this Wnt [41]. This critical serine residue is present in all Wnt, suggesting that the levels of 16:1n-7acylation may play a crucial role in regulation of Wnt signaling. It is currently unknown if this 16:1-n7 is largely originated from SCD activity or from exogenous sources. Therefore we examined the synthesis and secretion of Wnt7b and Wnt5a, two paradigmatic canonical and non-canonical Wnt proteins, respectively, in cells expressing SCD5 activity. To do so, we overexpressed either Wnt7b or Wnt5a genes tagged with V5 epitope in Neuro2a cells and the intracellular and secreted levels of these Wnt ligands were assessed by immunoblotting. The use of tagged constructs facilitates assessment of overexpressed Wnt ligands by using antibodies raised against the tag peptide without considering endogenous ligands. As shown in Figure 8A, SCD5-expressing cells showed reduced levels of Wnt7b with respect to vector-transfected controls. Compared to control cells, SCD5 cells secreted lower amounts of Wnt7b, regardless of differences in synthesis observed between control plasmid and SCD5-transfected cells. The finding that GAPDH, a cytosolic protein used as loading control in the immunoblot analysis, was not detected in media rules out that different levels of secreted Wnt were caused by cell leakage or by contamination during the preparation of samples. As a control for specificity of the signal we simultaneously analyzed non-transfected cells. We also analyzed cells transfected with the empty plasmid in which Wnt ligand construct was inserted. No signal was detected in cell lysates neither in supernatants. Importantly, we detected reduced cellular levels of Wnt5a but greater levels of this Wnt protein in the media fractions of SCD5-expressing cells than in their controls (Figure 8B), suggesting a modification of Wnt5a secretion caused by SCD5. The fact that when Sox17 and TCF cDNA were transfected in control and SCD5-expressing cells the levels of these two proteins were unmodified by the presence of SCD5 activity (data not shown) rules out a global effect of SCD5 activity on protein production. Altogether, these experiments suggest that, by altering the n-7 MUFA-to-SFA ratio, SCD5 activity differentially modulates the production and secretion of canonical and non-canonical Wnt ligands, which may account for the profiles in Wnt-induced signaling activity observed in SCD5-expressing cells.

**Figure 9:**
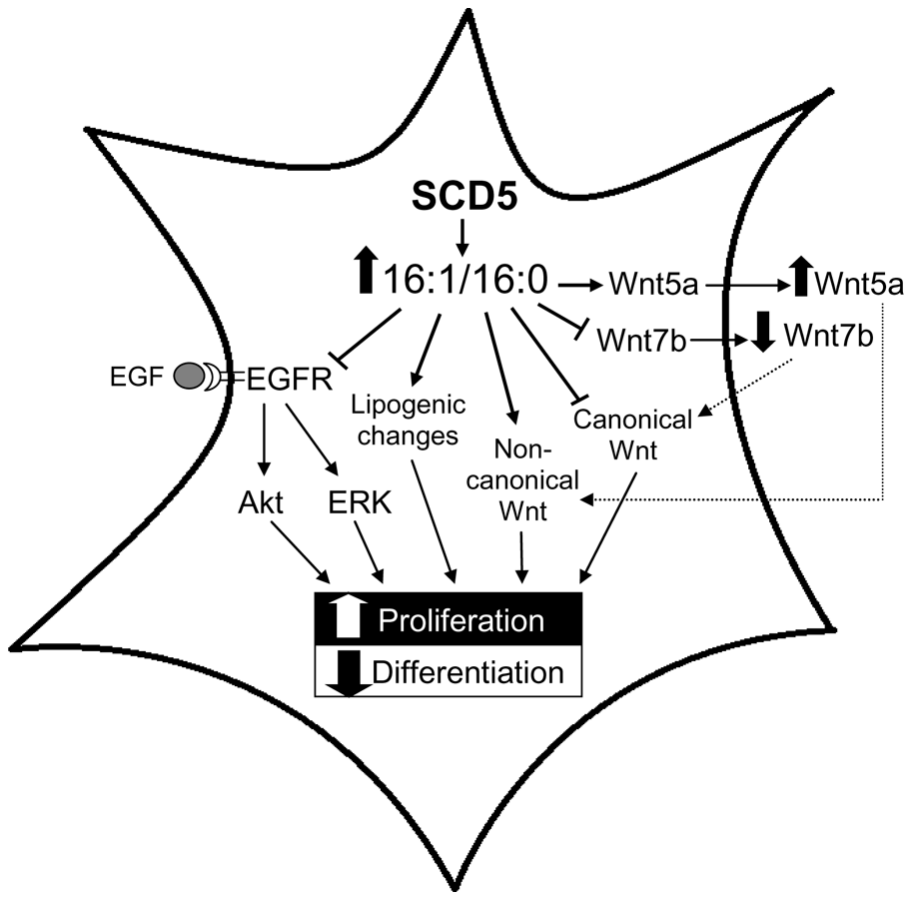
Hypothetical mechanisms by which SCD5 activity modulates proliferation and differentiation in neuronal cells. Elevated palmitoleic acid-to-palmitic acid (16∶1/16∶0) ratio promoted by SCD5 expression can contribute to high proliferation and low differentiation rates in neuronal cells through multiple mechanisms, including deactivation of EGFRAkt/ERK signaling pathways, alteration of lipogenesis, and divergent regulation of canonical and non-canonical Wnt pathways by affecting the secretion of Wnt ligands and/or the modulation of intracellular Wnt activity.

## Discussion

A complex synchronization of cell division, exit of cell cycle and differentiation is required for the proper development of the nervous system. The mechanisms of neuronal proliferation and differentiation involve an intricate array of biochemical and morphological changes that require a finely tuned modulation of signaling pathways and lipogenic routes. The particular pattern of expression of SCD5, with the highest levels in embryo and adult brain [21], [23], suggest that a potential role for SCD5 in the mechanisms of proliferation and differentiation of neural cells. Here we report that ectopic expression of SCD5 induces a drastic phenotypical modification in Neuro2a cells, which is characterized by a marked increase in the rate of cell replication and a drastic suppression in the formation of branching neurites, a terminal marker of the neuronal differentiation process.

We hypothesize that the greater rate of cell proliferation of SCD5-expressing cells could have been caused by an acceleration of cell cycle, a notion that is supported by the finding of much greater levels of cyclin D1, a crucial regulator of the G1/S transition in cell cycle progression [38], in these cells. Since exit from cell cycle is a prerequisite for the terminal differentiation of neurons [6], a more active cycle of cell replication could explain the delay, or even failure, of SCD5-expressing neuronal cells in fully developing into differentiated neurons. In this regard, we observed that induction of the differentiation program with retinoic acid markedly halted cell proliferation in both SCD5-expressing and controls cells, however the effect was less marked in the former cell group, indicating a more robust cell growth activity.

In addition, our studies indicate that, although SCD5 may be key factor in defining the biological fate of neuronal cells, the desaturase is itself not targeted by the differentiation program. This notion is supported by our findings that levels of SCD5 protein remained unchanged during the course of differentiation of SH-SY5Y human neuroblastoma cells with retinoic acid, and in similar incubations with retinoic acid in differentiated skin fibroblasts. That is to say, it appears that SCD5 does not lie downstream of the initiation in the pathway, but rather it independently modulates the differentiation pathway.

Modifications in critical biochemical and metabolic features in neuronal cells, such as the changes in acyl-lipid composition and lipid biosynthesis that were detected in Neuro2a cells expressing SCD5, could also contribute to the phenotypical perturbations promoted by this SCD variant. As expected for a Δ9 desaturase, expression of SCD5 increased the MUFA:SFA ratio in cellular lipids, although the enrichment of lipid with MUFA was restricted to MUFA belonging to the n-7 series, chiefly palmitoleic (16∶1) and cis-vaccenic (18∶1) acids. An intriguing observation in our studies was that the levels of oleic acid remained surprisingly unaltered in the SCD5-expressing Neuro2a cells, suggesting a preference of the desaturase for palmitic acid as substrate. Data from in vitro determinations clearly showed that SCD5 was able to desaturate both palmitic and stearic acid at approximately similar catalytic rates [22]. We believe that the particular modifications in fatty acid composition of SCD5-expressing cells could be attributed to cell type-specific fatty acid biosynthetic enzymes, such as differential elongase activity, since a similar SCD5 expression cell model generated in mouse 3T3-L1 preadipocytes significantly elevated the content of oleic acid (data not shown). In any case, the higher MUFA content observed in SCD5-expressing Neuro-2a cells may be functionally associated with their faster cell replication, since it has been established that dividing cells, both normal proliferating cells and cancer cells, have a critical dependence on endogenously synthesized MUFA for sustaining an active mitogenic program [15], [34]. Furthermore, in cancer cells in which SCD activity was pharmacologically blocked, addition of n-9 or n-7 MUFA were equally effective in restoring the cell proliferation rate [19], indicating that all MUFA exhibit pro-growth and pro-survival functions.

Besides modulation of the fatty acid profile of neuronal lipids, we found that SCD5 also is implicated in the control of glucose-mediated lipogenesis. We observed that SCD5 does not appear to globally stimulate lipid biosynthesis from glucose, but to promote a selective increase in the biosynthesis of phosphatidylcholine and cholesterolesters, in parallel to decreases in the production of phosphatidylethanolamine and triacylglycerol. This observation suggests that unlike SCD1, which appears to control the overall rate of lipogenesis in proliferating cells [34], SCD5 activity is functionally linked to more specific biosynthetic routes. Importantly, the alterations in lipid biosynthesis caused by SCD5 expression could also contribute to the drastic change in the rates of cell replication and differentiation observed in SCD5-expressing cells. Phospholipid synthesis, particularly phosphatidylcholine, is critically needed for propelling the progression of cell cycle [42], [43] and for avoiding the entry in the apoptotic program [44]. Therefore, the activation of the de novo phosphatidylcholine synthesis in cells expressing ectopic SCD5 may provide abundant lipid substrates for the formation of new cell membranes to support their accelerated rate of cell proliferation. The activation of phospholipid biosynthesis, especially phosphatidylcholine though the Kennedy pathway, is also a metabolic requirement for neurite outgrowth and branching [5], [45], [46]. We concede that in the present work, for reasons of scope and focus, we did not fully address the potential role of SCD5 activity in phospholipid formation during neuronal differentiation; a potential contribution of the desaturase to the regulation of this lipogenic pathway cannot be ruled out without experimental evidence yet to be established.

The coordination of proliferation and differentiation in neurons not only demands appropriate metabolic conditions but also the action of extrinsic signals, such as EGF, platelet-derived growth factor (PDGF), fibroblast growth factor 2 (FGF-2), nerve growth factor (NGF), and Wnt proteins, among other cytokines [2]-[6]. Depending on the phenotypical phase to which the neuronal cell is committed, these factors will trigger selective mechanisms that will activate cell proliferation when expansion of the neuronal population is required, or initiate the program of differentiation [6]. The growth factor-mediated activation of tyrosine-kinase receptors, as well as their downstream signaling effectors Akt, ERK and mTOR, are key for the execution of transcriptional mechanisms that leads to cell cycle arrest and morphological differentiation of neurons [2], [3], [6], [47]. Activation of EGFR, a paradigmatic receptor that is often associated with the mitogenic response in normal and cancer cells, is particularly critical for the execution of these biological events in neuroblastoma cells [48], [49] and in brain astrocytes [50]. Therefore, the suppression of neurite outgrowth in SCD5-expressing Neuro-2a cells could then be explained by the marked blockade in the EGF-induced phosphorylation/activation of EGFR and surrogate signals ERK and Akt observed in these cells when the program of differentiation was induced. Data from our group and others demonstrated that SCD1 activity is critical for the functional activation of Akt and ERK [16], [51]. Our observation that SCD5 negatively regulates these two signaling proteins upon growth factor stimulation suggests different roles for the human SCD isoforms in the modulation of signaling cascades. However, since our determinations were restricted to a neuronal cell line, the possibility of cell-type specific differences in the participation of SCDs in signaling regulation cannot be ruled out at the moment.

Our results also point out to Wnt proteins as a second signaling mechanism related to neuronal differentiation that is a target for SCD5 activity. Our data showing that SCD5 activity activates the non-canonical Wnt pathways while suppressing the canonical branch of Wnt are in agreement with a previous report of a role for the endogenous biosynthesis of MUFA in the regulation of Wnt signals in mouse skin [52]. These authors observed that lack of SCD1 expression led to a suppression in the expression of Lef1, a key transcription factor involved in the regulation of canonical Wnt/β-catenin signaling. Interestingly, ablation of Lef1 downregulates SCD1 expression in mouse skin [53], suggesting a positive activation loop between MUFA and Wnt activation. Since we found that, besides SCD1 [54], human skin fibroblasts express significant levels of SCD5, this SCD isoform may also have a role in the regulation of Wnt signaling in human skin tissues.

Perturbations in Wnt signaling pathways caused by ectopic SCD5 activity may be responsible for some of the effects in the processes of proliferation and neurite outgrowth in neuronal cells communicated in this report since a number of studies, albeit conflicting, have demonstrated the critical role of Wnt signaling on neurogenesis and neuronal differentiation. In neural precursor cells and in the developing neocortex of mice, the rate of neuronal differentiation was positively associated to the degree of activation of the canonical Wnt pathway [8], but in neuronal cells canonical Wnt have been also shown to increase neuronal proliferation and to suppress differentiation [55]–[57]. The discrepancies in the function of Wnt in cell proliferation and differentiation of neuronal cells could also arise from the fact that Wnt may activate or deactivate both events in a time- and stage-specific manner [7], [39]. Also importantly, depending on the physiological context of the neuronal cells, a Wnt ligand may elicit either canonical or non-canonical in these cells [58], [59].

The complexity of the relationship between biological processes like neurogenesis and neuritogenesis and Wnt activity is further highlighted by the fact that posttranslational modifications of Wnt proteins profoundly affect their functional properties. For instance, Wnt-3a is targeted for S-palmitoylation in its Cys77 residue, an event that is critical for signaling activation [41]. More recently, Galli and Burrus [60] reported that acylation of Wnt1 in specific residues affects both Wnt signaling branches, showing that binding of palmitoleic acid to S224 preferentially signals via the ß-catenin pathway while acylation with palmitic acid (palmitoylation) on C93 primarily impacts the non-canonical, ß-catenin-independent pathway.

Our experiments provide evidence that SCD5 modulates not only the activity of Wnt signaling pathways but also the levels of secreted of Wnt ligands, suggesting an additional regulatory mechanism by which the desaturase may control cell proliferation, differentiation, or both. The observation that SCD5 activity enhances the secretion of non-canonical Wnt5a while it reduces the synthesis and, consequently, the secretion of canonical Wnt7b may partly explain the finding of similar changes in the activity in both branches of Wnt signaling in SCD5-expressing cells. The mechanisms by which SCD5 activity controls the production and release of Wnt are currently unknown. Synthesis, intracellular transport and secretion of Wnt are tightly regulated events that also depend on posttranslational modification by fatty acid acylation. Through a battery of elegant experiments, Takada et al. [41] demonstrated that Wnt-3a is specifically acylated on Ser209 with palmitoleic acid (16:1 n-7) and that this modification is essential for the intracellular transport and secretion of this Wnt. These authors reported that Ser209 is part of an amino acid sequence that is highly conserved among the members of the Wnt family, suggesting that other Wnt proteins may be modified, structurally and functionally, by palmitoleyl acid (16:1 n-7) acylation. In most acylated proteins, an unsaturated fatty acid could conceivably be replaced by a saturated fatty acid in a residue targeted for acylation, hence in SCD5-expressing cells the abundance of palmitoleic acid may displace palmitic acid from the acylation site in Wnt proteins, potentially modifying their transport, their rate of secretion, or both. However, although it is clear that SCD5 expression alters the homeostatic control of Wnt proteins, at this early stage in the investigation, the questions of whether SCD5-mediated modification of Wnt synthesis and secretion is related to a potential change in their acylation, and whether changing levels of Wnt are responsible for the alterations in the activation of Wnt pathways in SCD5-expressing cells await further experimental confirmation. Finally, given the mounting evidence suggesting a mechanistic association of abnormal Wnt signaling with Alzheimer's and Parkinson's diseases [61], our findings suggest that SCD5 may be a molecular link between signaling and lipogenic pathways mechanisms and these neurodegenerative conditions.

In conclusion, the present study provides the first evidence that human SCD5 activity is implicated in the regulation of critical biological functions in neuronal cells. Our findings imply that, by modulating fatty acid composition, lipogenesis and intracellular signaling, SCD5 controls the rate of replication and differentiation of neuronal cells (Figure 9). We observed that the constitutive expression of human SCD5 promotes a shift in the fatty acid composition in lipids of neuronal cells, which was characterized by elevated levels of n-7 MUFA with a concomitant reduction in SFA. These modifications in the fatty acid pattern were accompanied by lipogenic alterations, such as the change in the rate of synthesis of phospholipids, which are known to affect cell growth, survival and differentiation. Remarkably, SCD5 expression promotes a profound deregulation of EGF's intracellular signaling mechanisms. We observed that SCD5 expression suppresses the ligand-induced activation of the EGFRAkt/ERK signaling platform. SCD5 activity also reduces the activation of canonical Wnt signaling whereas it stimulates the non-canonical branch of the Wnt pathway. These activity changes could be directly related to the perturbations in the synthesis and secretion of Wnt proteins observed in SCD5-expressing neuronal cells. We also found that SCD5 expression accelerates cell cycle progression while suppressing the program of differentiation, indicating that the fate of neuronal cells is, ultimately, determined by the activity of the desaturase. Lastly, our studies suggest a value for SCD5 as a potential target for clinical interventions in poorly-treated neurological diseases such as brain cancer, Alzheimer and other neurodegenerative conditions.
